# The study of halogen effect on the reactivity of the serine-targeting covalent warheads

**DOI:** 10.3389/fchem.2024.1504453

**Published:** 2024-12-03

**Authors:** Conghao Gai, Ya Zhang, Shihao Zhang, Xueyan Hu, Yun-Qing Song, Xiaoyu Zhuang, Xiaoyun Chai, Yan Zou, Guang-Bo Ge, Qingjie Zhao

**Affiliations:** ^1^ Organic Chemistry Group, College of Pharmacy, Naval Medical University, Shanghai, China; ^2^ Shanghai Frontiers Science Centre of TCM Chemical Biology, Institute of Interdisciplinary Integrative Medicine Research, Shanghai University of Traditional Chinese Medicine, Shanghai, China

**Keywords:** hCES1A, covalent warheads, 2,2,2-trifluoroacetophenones, serine-targeted reactivity, halogen effect

## Abstract

Halogens favorably contributes to the drug potency and metabolic stability via electrostatic interactions. Herein, the halogen effects on the reactivity of the halogenated 2,2,2-trifluoroacetophenones as serine-targeting covalent warheads were investigated. Our results showed that introducing halogen atoms, especially Cl or Br, into the phenyl scaffold would influence the electron density around the ring, which led to different time-dependent inhibition response to the target serine hydrolase (hCES1A). Co-crystallography analysis not only verified that halogenated molecules preferred to form covalent adducts, but also provided the conformational information for the design of covalent inhibitors targeting to hCES1A protein for the treatment of drug-induced acute enteritis.

## Introduction

Halogens are frequently used in drug design and development. (Benedetto Tiz et al., 2022). Recent discoveries confirmed the multiple beneficial effects of halogens in the formation of drug-target complexes, such as lipophilicity. (Hernandes et al., 2010). Notably, incorporating a heavy halogen atom (bromine or iodine) into each compound would also further allow unambiguous identification of the position and orientation of the fragments by detection of a signal in X-ray crystallography that arises from the anomalous dispersion of the halogen atom. (Wood et al., 2019). The halogen bonds in ligand-target complexes favourably contributes to the drug potency and metabolic stability via electrostatic interactions as well. (Politzer et al., 2010; Brinck and Borrfors, 2019). However, it remains to be verified whether halogens may promote the formation of covalent adducts by the influence of changing the sensitivity of electrophilic warheads in covalent drugs.

## Rationale

To deeply understand the halogen effects on the covalent binding mechanism in ligand-protein pairs, a household library of fragments of small size and complexity, equipped with reported serine-selective electrophilic warheads such as sulfuryl fluoride, trifluoroacetyl and *β*-lactam, was constructed by us. (Martin et al., 2019; Narayanan and Jones, 2015) Confidence in this concept was provided by FragLite about building an efficient library of molecular fragments for fragment-based drug discovery. (Keeley et al., 2020; Lu et al., 2021). We innovatively applied this strategy in covalent fragment design. Human carboxylesterase 1A (hCES1A) was employed as a target enzyme for inhibitory activity (IC_50_) screening due to its conservative catalytic serine residue.

**FIGURE 1:**
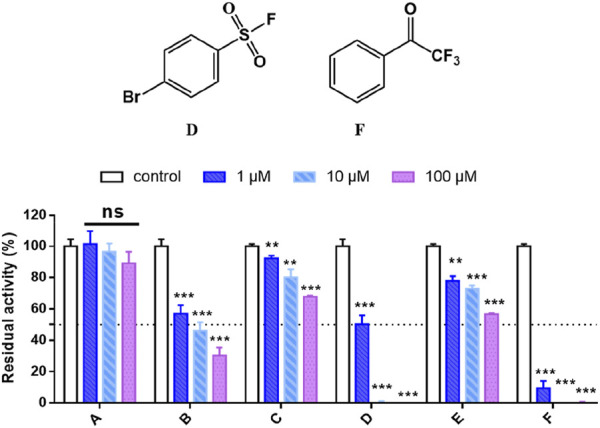
High-throughput screening of the household library of the covalent fragments, compounds D and F exhibit strong and selective inhibition against hCES1A. Data are expressed as mean ± SD and the results are analysed using t-test. *P < 0.05, **P < 0.01, ***P < 0.001 vs. control group.

As shown in Figures 1, 4-bromobenzenesulfonyl fluoride D and 2,2,2-trifluoroacetophenone F showed potent anti-hCES1A effects. Especially, as illustrated in Supplementary Figure S1, F exhibited 2-folds better potency after 33 min incubation (IC_50_ = 11 nM) against hCES1A but no significant time-dependent inhibition against hCES2A, which in degree reflects the reality that 2,2,2-trifluoroacetophenone selectively forming weak covalent interactions with the serine of hCES1A. Then, fragments D and F were also screened to validate their selectivity towards a series of serine-related targets, including hCES1A, hCES2A, Notum, and hPL. D and F both showed decent selective inhibitory activities on hCES proteins at a dose concentration of 10 μM (Supplementary Figure S2). But, there was no potent responses to Notum and hPL, indicating that hCES proteins are more sensitive to the sulfuryl fluoride and the trifluoroketone warheads. Considering that the IC50 values of hCES1A and hCES2A for compound D exhibited no statistically significant difference (Supplementary Figure S3), we chose F as a starting fragment for further halogenation around the phenyl ring.

**FIGURE 2:**
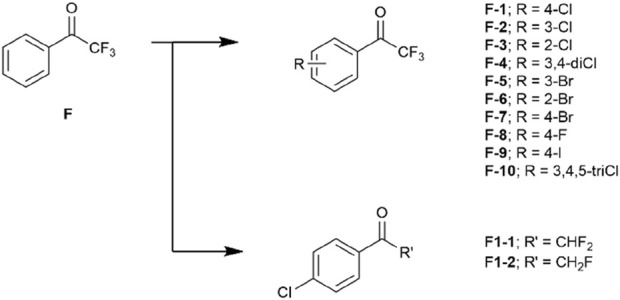
The halogenated fragment library derived from trifluoroacetophenone F.

A drug-like fragment library including a series of halogenated trifluoroacetophenones were then obtained (Figure 2). We also prepared compounds F1-1 and F1-2 to investigate the necessary of trifluoromethyl effect on covalent bond formation (Figure 2).

## Results and discussion

Initially, computational chemistry was used to predict the covalent bonding mode of trifluoroacetyl group that proceeds the nucleophilic attack of the carbonyl carbon by the hydroxyl group of serine. The observed differences in reactivity were rationalised from the calculated energy gaps between the trifluoroacetyl LUMO and the ‘capped’ methyl acetylserinate HOMO (Supplementary Table S1). Calculations employed the rDFT/6-31+G(d,p) level of theory in the Gaussian09 suite of programs (Frisch et al., 2024). Geometries were optimised and frequencies computed to verify that they are minimal.

**FIGURE 3:**
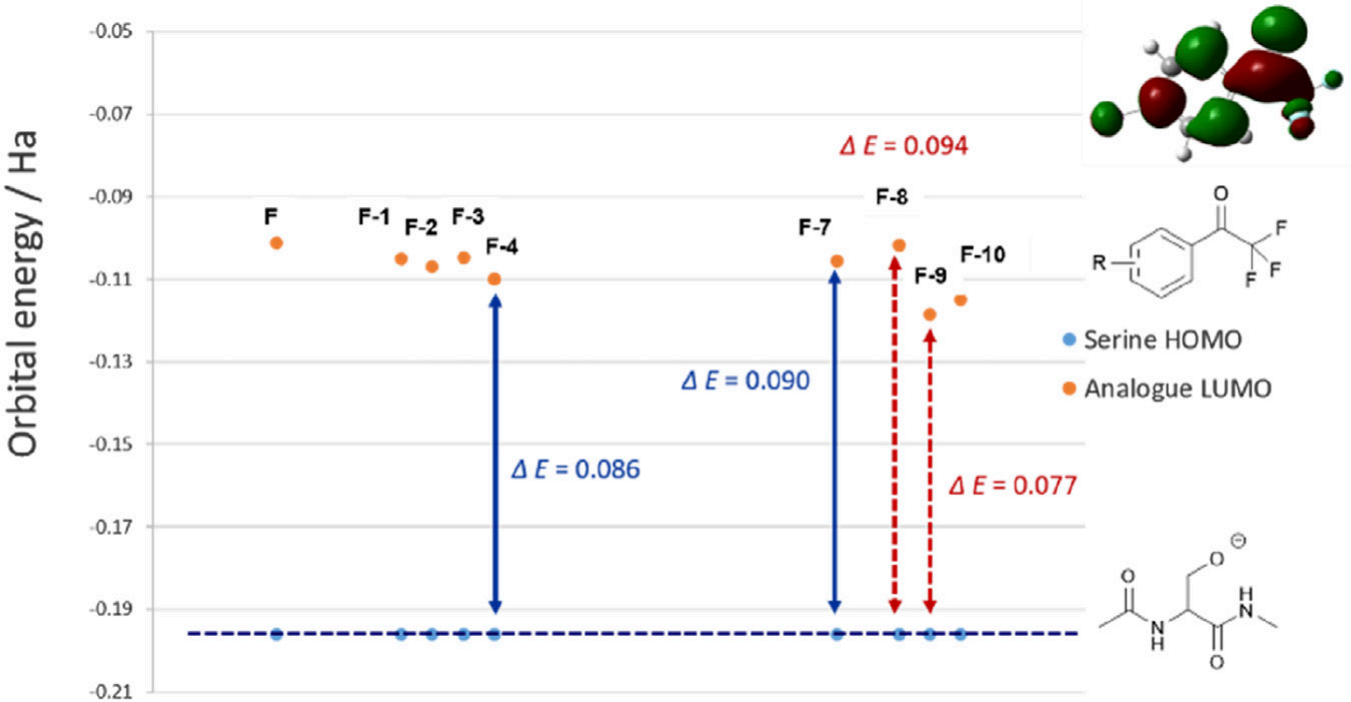
Calculated HOMO energies at the rDFT/6-31+G (d,p) level for ‘capped’ serine relative to the halogenated trifluoroacetophenone analogues LUMO, within the errors of the basis set. Double-headed arrows show the average HOMO–LUMO gaps.

Results of the virtual screening provides a rationale as to why the halogen substituents may promote inhibitions. LUMOs of the chloro-species are consistently lower in energy than the parent compound F (Figure 3). This enlightens that introducing chloride atom into the phenyl ring would theoretically provide obvious time-dependent inhibition as the HOMO–LUMO energy gap is significantly reduced (*ΔE* (F) = 0.095 Ha, *ΔE* (F-4) = 0.086 Ha). HOMO of fluoro-substituted analogue F-8 is higher in energy compared to other halogenated species (HOMO–LUMO *ΔE* = 0.094 Ha), which suggests the loss in potency to hCES1A because of this big bonding energetic gap. 4-I analogue F-9 exhibits the lowest LUMO energy, which potentially provides the best inhibitory activity against hCES1A enzyme (Figure 3).

Then, *in vitro* assays are set up to verify the virtual screening results. The IC_50_ values against hCES1A at 3 min and 33 min, respectively, were listed in Table 1. It was encouraging to see that introducing halogen atoms into the phenyl ring was all tolerated, which highly improves time-dependent inhibitory activities against the target as we expected. Notably, if replacing the halogens into other polarised groups such as OMe and aminos, molecules lost their time-dependent inhibitory activities to hCES1A, which verified the significance of halogen effect from the side (Supplementary Table S3).

**TABLE 1:** Inhibitory activities of the halogenated fragment library against hCES1A.^a^

Cmpd	R			R’	hCES1A IC_50_ (nM)		
	4-	3-	2-		3 min	33 min	Fold Increase^b^
F	H	H	H	CF_3_	24.1 ± 1.0	11.1 ± 2.0	2.2
F-1	Cl	H	H	CF_3_	14.4 ± 1.9	1.9 ± 0.2	7.6
F-2	H	Cl	H	CF_3_	34.1 ± 5.3	2.5 ± 0.4	13.6
F-3	H	H	Cl	CF_3_	11.6 ± 0.7	2.3 ± 0.4	5.0
F-4	Cl	Cl	H	CF_3_	10.0 ± 1.4	2.4 ± 0.4	4.2
F-5	H	Br	H	CF_3_	17.0 ± 3.0	1.7 ± 0.5	10.0
F-6	H	H	Br	CF_3_	9.9 ± 1.01	52.8 ± 15.9	0.2
F-7	Br	H	H	CF_3_	16.1 ± 2.3	2.1 ± 0.6	7.7
F-8	F	H	H	CF_3_	1,703.0 ± 373.7	602.6 ± 107.1	2.8
F-9	I	H	H	CF_3_	37.7 ± 8.6	6.2 ± 1.1	6.1
F-10	Cl	Cl	Cl	CF_3_	30.0 ± 4.5	2.8 ± 0.4	10.7
F1-1	Cl	H	H	CHF_2_	58.0 ± 6.0	101.6 ± 37.4	0.6
F1-2	Cl	H	H	CH_2_F	1,206.0 ± 46.0	873.9 ± 92.2	1.4

^a^ Data are reported as the mean ± SD (n = 3 repeats).

^b^ The ratio retains two significant digits.

As expected, the IC_50_ values of 4-substituted analogues presented a gradient correlation with the electronegativity around the halogen atoms. We gladly see that 4-fluoro analogue F-8 does not inhibit hCES1A. This is consistent with the results predicted by virtual screening. Work on the 4-position of the ring concluded that introducing a fluoride atom abolishes potency whereas replacing it with a chloride or bromine atom is roughly equipotent. When the 4-position was replaced by an iodine atom, analogue F-9 shows better anti-hCES1A activity than the parent compound F after 33 min incubation, even though this was not prominent at 3 min. This perhaps implied that the iodine atom was too bulky to dock analogue F-9 into the catalytic pocket, leading to the intolerance of the ligand within active region of the protein which caused weak inhibition after 3 min incubation. Surprisingly, dichloro-substituted analogues F-4 deriving from analogues F-1 and F-2 exhibited more promising contribution than monosubstituted analogues, but introducing three chloride atoms into the ring was not tolerated perhaps due to their enhanced passivation effect. According to comparative results between F-1 and F1-1/-2, trifluoromethyl group is necessary for the covalent inhibition as expected, rationally promoting the electrophilicity of carbonyl carbon sensitive to the nucleophilic attach by the serine residue.

Although the poor anti-hCES1A effect may be due to molecular conformation intolerant to the protein pocket, the covalent binding ability of the trifluoroacetyl warhead of halogenated analogues could be roughly reflected by the results of time-dependent inhibition assays. As shown in Table 1, the parent compound F exhibits weak time-dependent inhibition against hCES1A. It is encouraging to see that all halogens extremely promote TDIs. Especially, both 4-Cl and 3-Cl substituted analogues display near 10-folds inhibition at 33 min better than that at 3 min. This interesting result highlighted the fact that halogens may increase the electron-withdrawing capacity of aromatic rings through their inductive effect, thus improving the electrophilicity of carbonyl carbon and making it more electron-deficient easily be attacked by the hydroxyl group of serine.

The covalent interactions were evaluated by nano LC-MS/MS, which offered powerful evidence to support the formation of the covalent adducts and well-explained the time-dependent inhibition of these covalent fragments. We also aim to use these probes to search for potential covalent binding sites on the target protein. Chloro-species compounds F-3 and F-4 were chosen to detect their covalent binding sites on hCES1A (Supplementary Table S2). Since the activity of electrophilic warheads and the formation of covalent adducts are related to temperature, we set the incubation conditions at two different temperature points (4 °C and 25°C, respectively). This analysis certainly found several allosteric binding sites (Supplementary Table S2), although no expected result of any compound covalently binding to Ser221 at the catalytic cavity of hCES1A (Supplementary Figures S4, S5).

**FIGURE 4:**
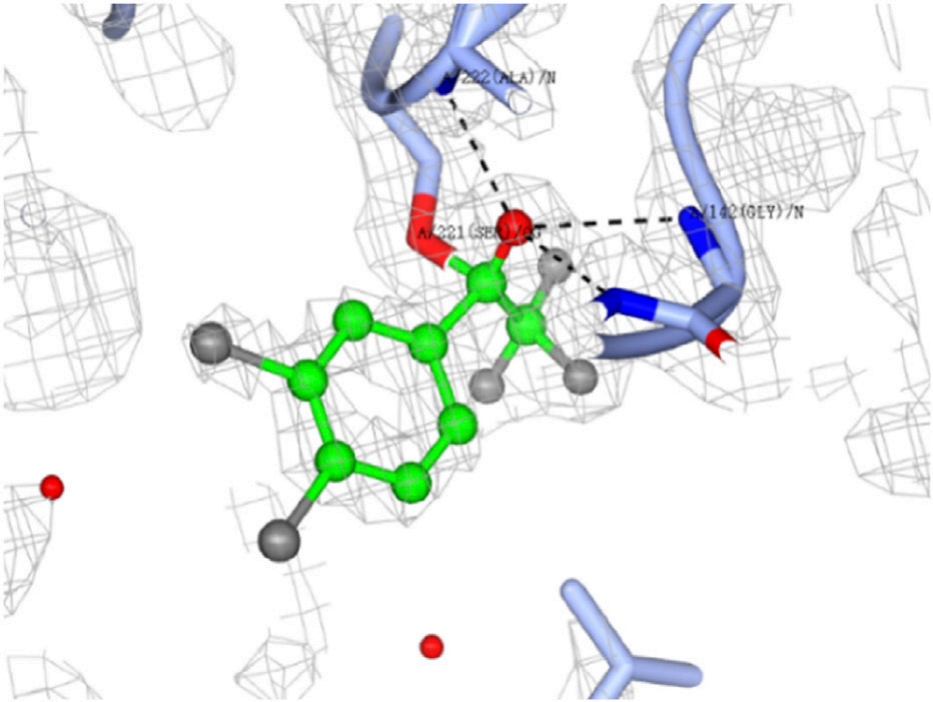
Dose-response crystal soaks of 3.4-dichloro analogue F-4 (green ball and stick) into hCES1A (ice blue ribbons). Electron density of bound ligand is shown in grey mesh. Hydrogen bonding interactions are highlighted in black dotted lines. Water molecules are shown as red spheres. Structure determined by Dr Shilong Fan (Technology Center for Protein Sciences, Tsinghua University) at a resolution of 1.83 Å. The image generated using CCP4MG.

The hCES1A binding site is the specific region on the enzyme where molecules, such as drugs or substrates, can bind and interact. Understanding the nature of the target and its binding site in terms of druggability is crucial for developing effective therapeutics. By the analysis of X-ray crystal structure, human CES1A has a serine esterase catalytic triad (Ser-221, His-468, and Glu-354), such catalytic triad is conserved in all mammalian species. (Arena de Souza et al., 2015; Song et al., 2021). Successfully, the co-crystallography of hCES1A with F-3 and F-4 provided promising covalent binding modes (Figure 4) at the catalytic triad. It could be obviously seen that the carbonyl group of F-4 was attacked by the oxygen of the OH group of Ser221, forming an expected hemiacetal covalent bond (Figure 4). The OH of the hemiacetal group introduced multiple hydrogen interactions with Gly143, Gly142 and Ala222 nearby, stabilising the formed hemiacetal adduct and favouring binding of the compound at this site (Figure 4). Notably, the co-crystallisation of the parent compound F and hCES1A failed to result in similar covalent interactions. This further proves that the halogen effect may promote the formation of covalent adducts by changing the sensitivity of the electrophilic warhead.

## Experimental

### Chemistry

The household library of covalent fragments including D and F was from Organic Chemistry Group, NMU. Commercially available materials were ordered from commercial suppliers (Bidepharm, Shanghai, China) and used without further purification. Structures were checked by ^1^H- and ^13^C-NMR spectra on a Bruker AVANCE 600/125 MHz instrument for confirmation, using DMSO-*d*_6_ or CDCl_3_ as the solvent. Molecular orbitals were calculated using Gaussian 09W and image generated by GaussView 6.0. (Frisch et al., 2024).

### Inhibition assay on hCES1A

In this study, a known bioluminogenic probe substrate NLMe was used for human carboxylesterase 1 (hCES1A) inhibition assays. (Wang et al., 2020). In brief, the incubation mixture (0.1 mL) consisted of 0.1 M buffer PBS (PH 6.5), hCES1A (0.1 μg/mL, final concentration) and each test compound. Preincubated for 3 min at 37°C and then NLMe (20 μM, final concentration) was added to initiate the hydrolytic reaction. After 20 min incubation, equal volume of LDR was added to stop reaction. The luminescence signals were monitored in the microplate reader (SpectraMax iD3Molecular Devices, Austria).

### Mass spectrometry peptide analysis

The F-3, F-4-bound peptides was analysed using the nanoLC-MS/MS system, respectively. (Fang et al., 2015). The purified hCES1A (150 μg, final content) was co-incubated with the small molecule inhibitor F-3/F-4 (100 μM, final concentration) at 4 C and 25°C, respectively, for 4 h. Then to finish the process of protein denaturation, reduction, and alkylation, the mixtures were treated with urea, DTT, and IAA. Following this, the enriched proteins were solubilised in NH_4_HCO_3_ solution (pH 8.0, 50 mM) containing 5% ACN, and then chymotrypsin and trypsin were used to digest the proteins at 37 °C for 16 h. After overnight incubation, the digestion was terminated with 15 μL 10% FA. The resulting peptides were desalted on a MonoSpin C18 column (GL Sciences Inc.). After the eluents was dried, it was redissolved with 20 μL 0.1% FA for test.

### Crystallization and structure determination

Purified hCES1A incubated with F-3 and F-4 at 1:5 M ratio for 1 h at 18°C. Crystallisation screening was performed using commercially available kit sets using sitting drop vapor diffusion at 16°C. Both diffraction-quality crystals were obtained from the condition containing 14.4% peg8000 (w/v), 0.1M MES 6.4, 0.3M CaAc and 20% glycerol. Crystals were flash cooled in liquid nitrogen after cryoprotection in 25% glycerin. Date was collected on 02U1 at Shanghai Synchrotron Radiation Facility (SSRF). Data images were processed using program HKL2000. (Otwinowski and Minor, 1997). Crystal structures were solved using molecular replacement with the Phenix software suite using unbound hces1a (Protein Data Bank [PDB] accession number 1MX1 as the search model. Structure refinement was performed using Phenix. (Adams et al., 2010). The program COOT was used for manual rebuilding. (Emsley and Cowtan, 2004). Molecular graphics images were generated using CCP4MG. (McNicholas et al., 2011).

## Conclusion

Trifluoroacetophenone warhead exhibits promising covalent binding activity which highly correlates with its chemoattractant-like electron-poor carbonyl group. While, difluoroacetophenone and monofluoroacetophenone hardly produce covalent bonds with the serine residue. Moreover, stronger halogen bonds can enhance the binding affinity between drugs and target proteins, thereby increasing the drug’s activity and effectiveness. We could see that introducing halogen atoms into the phenyl scaffold indeed influences electron density around the ring leading to different time-dependent inhibitory response of ligands to the enzyme. Chloro- and bromo-substituted analogues appear to the most tolerated species. This is because halogen atoms have high electronegativity and can form strong interactions with the backbone of the target protein. Therefore, the stronger the halogen bond, the tighter the binding between the ligand and the target protein, resulting in stronger inhibitory activities.

In addition, the polarisability of halogen atoms may also impacts covalent drug interactions. Halogen atoms with higher polarisability can induce stronger polarisation effects when covalently interacting with polar groups in the target protein. The induced polarisation effect can promote the covalent bond formation between the electrophilic warhead and the nucleophilic residue in target proteins, thereby enhancing the drug’s activity.Due to the aforementioned reasons, the formation of a covalent adduct utilising trifluoroacetophenone is strongly correlated with its electron-deficient carbonyl group, which exhibits chemoattractant-like properties. The introduction of halogen atoms, particularly chlorine, into the phenyl scaffold enhances the benzocarbonyl reactivity in forming hemiacetal adducts with serine.

Covalent molecules have the potential to form strong and stable bonds with their biological targets, making them appealing for various therapeutic applications. However, their reactivity can lead to off-target interactions, causing unintended side effects. Achieving target selectivity is crucial to minimize off-target effects and enhance the safety profile of covalent molecules. This requires careful design and optimization of the molecules to ensure their specific binding to the intended target while minimizing off-target interactions with other proteins or biomolecules. These trifluoroacetophenone hits selectively bind to hCES1A and display weak inhibition against the homologous protein hCES2A. Therefore, leveraging on the successful co-crystal structures, our next endeavour will focus on deriving covalent lead compounds that selectively target the hCES1A protein for the anti-inflammatory researches.

## Data Availability

The datasets presented in this study can be found in online repositories. The names of the repository/repositories and accession number(s) can be found in the article/Supplementary Material.
