# Supplementary material for: The study of halogen effect on the reactivity of the serine-targeting covalent warheads
Source: Front Chem. 2024 Dec 3;12:1504453. doi: 10.3389/fchem.2024.1504453 (PMC11649397; doi:10.3389/fchem.2024.1504453)
Supplement: Supplementary file 1 [file DataSheet1.pdf]

Supplementary Information

The unique role of halogen substituents on the reactivity of the serine-targeting covalent warheads

Conghao Gai,[#a] Ya Zhang,[#b] Shihao Zhang,[a] Xueyan Hu,[b] Yun-Qing Song,[b] Xiaoyu Zhuang,[b] Xiaoyun Chai,[a] Yan Zou,[a] Guang-bo Ge,[*b] Qingjie Zhao[*a]

[a] Organic Chemistry Group, College of Pharmacy, Naval Medical University, Shanghai, P. R. China, 200433, E-mail: c.gai2@outlook.com

[b] Shanghai Frontiers Science Centre of TCM Chemical Biology, Institute of Interdisciplinary Integrative Medicine Research, Shanghai University of Traditional Chinese Medicine, Shanghai, P. R. China, E-mail: geguangbo@shutcm.edu.cn

* Joint corresponding authors to this paper.

[#] Joint first authors to this paper.

Content

1. Detailed experimental, materials, and methods 2

Chemistry 2

Inhibition assay on hCES1A 2

Mass spectrometry peptide analysis 3

Crystallization and structure determination 3

2. Figures and tables 4

3. Reference 12

1. Detailed experimental, materials, and methods

Chemistry

The household library of covalent fragments including **D** and **F** was from Organic Chemistry Group, NMU (Fig. S1).

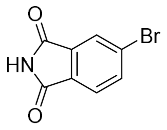

a

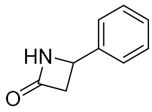

b

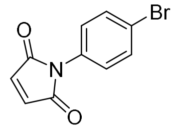

c

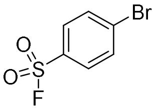

d

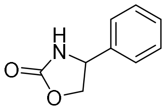

e

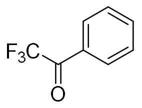

f

Fig. S1. Partial fragment molecules of a household library targeting to serine.

Commercially available materials were ordered from commercial suppliers (Bidepharm, Shanghai, China) and used without further purification. Structures were checked by ^{1}H- and ^{13}C-NMR spectra on a Bruker AVANCE 600/125 MHz instrument for confirmation, using DMSO- d_6 or $CDCl_3$ as the solvent. Molecular orbitals were calculated using Gaussian 09W and image generated by GaussView 6.0.[1]

Inhibition assay on hCES1A

In this study, a known bioluminogenic probe substrate NLMe was used for human carboxylesterase 1 (hCES1A) inhibition assays.[2] In brief, the incubation mixture (0.1 mL) consisted of 0.1 M buffer PBS (PH 6.5), hCES1A (0.1 µg/mL, final concentration) and each test compound. Preincubated for 3 min at 37 °C and then NLMe (20 µM, final concentration) was added to initiate the hydrolytic reaction. After 20 min incubation, equal volume of LDR was added to stop reaction. The luminescence signals were monitored in the microplate reader (SpectraMax iD3Molecular Devices, Austria).

Inhibition assay on hCES2A

Human carboxylesterase 2 (hCES2A) inhibition assays were conducted using FD, a known fluorescent substrate.[3] Briefly, hCES2A (0.4 µg/mL, final concentration) was preincubated with analytes/DMSO (as a control group) in PBS (pH 7.4) at 37 °C for 3 min. This was followed by adding the substrate FD (4 µM, final concentration) in the incubation mixture and allowing it to react for 30 min. The fluorescent signals (excitation/emission, 480 nm/525 nm) were continuously recorded by a microplate reader (SpectraMax iD3Molecular Devices, Austria).

Inhibition assay on human pancreatic lipase (hPL)

Human pancreatic lipase (hPL) inhibition assays were conducted using DDAO-ol, a reported

fluorogenic probe.[4] Briefly, the incubation mixtures (0.1 mL) contained Tris-HCl buffer, hPL solution (0.25 μg/mL, final concentration), porcine bile salt (0.1 mg/mL, final concentration) and each inhibitor (with different concentrations). After preincubated at 37 °C for 3 min, DDAO-ol (20 μM, final concentration) was added to initial reaction. The fluorescent signals of hydrolytic product were quantified by using the microplate reader (Spectra Max M4, Austria), with the wavelength of 600 nm and 660 nm for excitation and emission, respectively.

Inhibition assay on Notum

OPTS, a known fluorogenic probe, was used as the substrate to assess the residual activities of Notum in the presence of each tested compound.[5] Briefly, a mixture of 0.2 mL incubation system composed of Notum (0.01 μg/mL, final concentration), 0.1 M citrate phosphate buffer (pH 7.4), and each inhibitor (with different concentrations). After preincubated at 37 °C for 1 min, OPTS was added to initiate the hydrolytic reaction and the multi-mode microplate reader (SpectraMax iD3, Molecular Devices, Austria) was used to analyze the florescence signals of the hydrolytic product.

Mass spectrometry peptide analysis

The **F-3**, **F-4**, **F-10**-bound peptides was analysed using the nanoLC-MS/MS system, respectively.[6] The purified hCES1A (150 μg, final content) was co-incubated with the small molecule inhibitor **F-3**/ **F-4**/ **F-10** (100 μM, final concentration) at 4 ^{o}C and 25 ^{o}C, respectively, for 4h. Then to finish the process of protein denaturation, reduction, and alkylation, the mixtures were treated with urea, DTT, and IAA. Following this, the enriched proteins were solubilised in NH_4HCO_3 solution (pH 8.0, 50 mM) containing 5% ACN, and then chymotrypsin and trypsin were used to digest the proteins at 37 ^{o}C for 16 h. After overnight incubation, the digestion was terminated with 15 μL 10% FA. The resulting peptides were desalted on a MonoSpin C18 column (GL Sciences Inc.). After the eluents was dried, it was redissolved with 20 μL 0.1% FA for test.

Crystallization and structure determination

Purified hCES1A incubated with **F-3** and **F-4** at 1:5 molar ratio for 1h at 18 ^{o}C. Crystallisation screening was performed using commercially available kit sets using sitting drop vapor diffusion at 16 ^{o}C. Both diffraction-quality crystals were obtained from the condition containing 14.4% peg8000 (w/v), 0.1M MES 6.4, 0.3M CaAc and 20% glycerol. Crystals were flash cooled in liquid nitrogen after cryoprotection in 25% glycerin. Date was collected on 02U1 at Shanghai Synchrotron Radiation Facility (SSRF). Data images were processed using program HKL2000.[7] Crystal structures were solved using molecular replacement with the Phenix software suite using unbound hces1a (Protein Data Bank [PDB] accession number 1MX1 as the search model. Structure refinement was performed using Phenix.[8] The program COOT was used for manual rebuilding.[9] Molecular graphics images were generated using CCP4MG.

2. Figures and tables

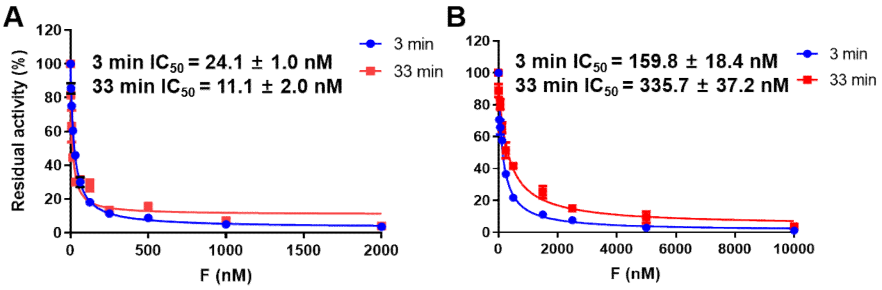

Fig. S1. Inhibitory activities of **F** against hCES1A (A) and hCES2A (B), respectively. Data are reported as the mean ± SD (n = 3 repeats).

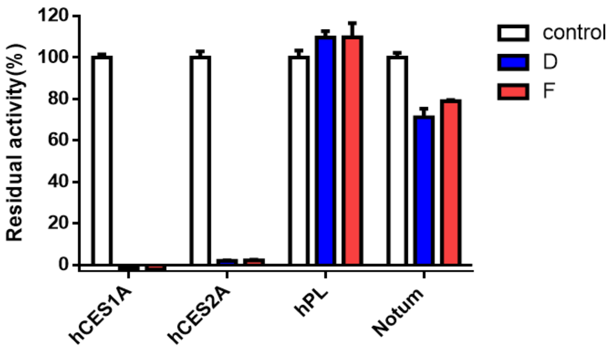

Fig. S2. Inhibitory selectivity screening of **D** and **F** (10 µM, final concentration) against hCES1A, hCES2A, hPL and Notum. Data are reported as the mean ± SD (n = 3 repeats).

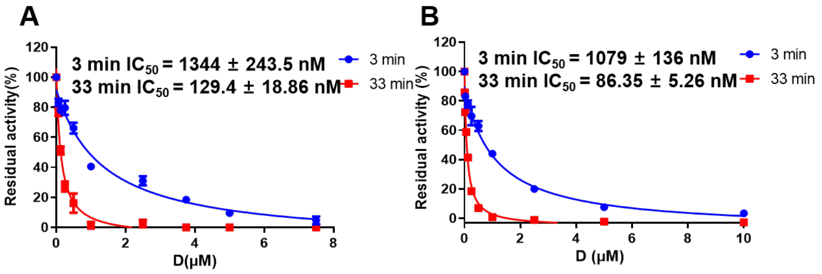

Fig. S3. Inhibitory activities of **D** against hCES1A (A) and hCES2A (B), respectively. Data are reported as the mean ± SD (n = 3 repeats).

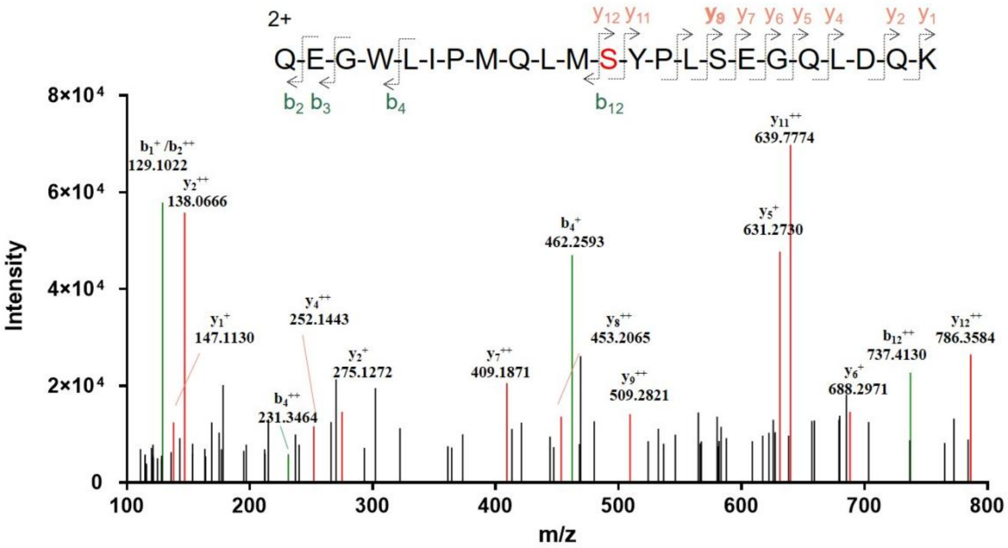

Fig. S4. The MS^2 spectra of the peptides containing QEGWLIPMQLMS^{365}YPLSEGQLDQK-stretch covalently modified by **F-3**.

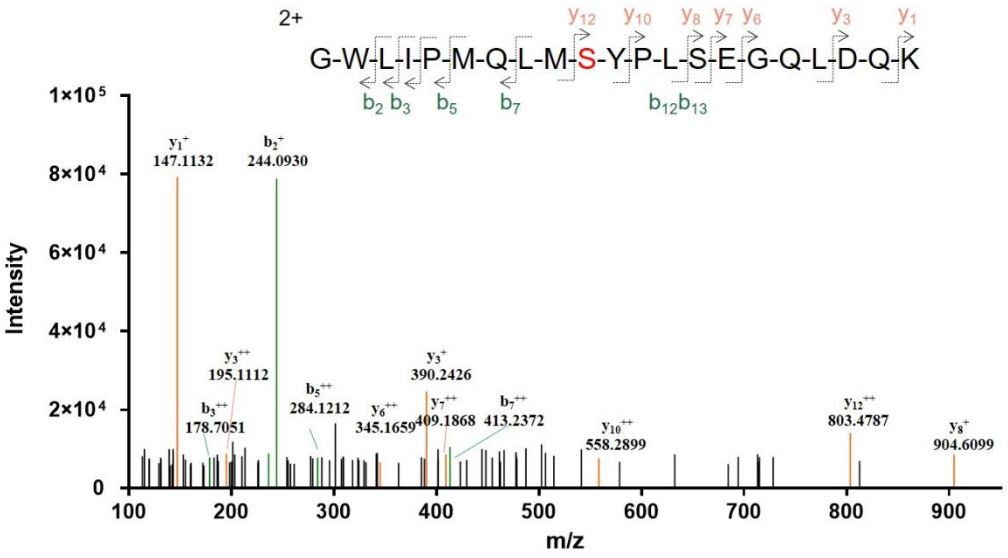

Fig. S5. The MS^2 spectra of the peptides containing GWLIPMQLMS^{365}YPLSEGQLDQK-stretch covalently modified by **F-4**.

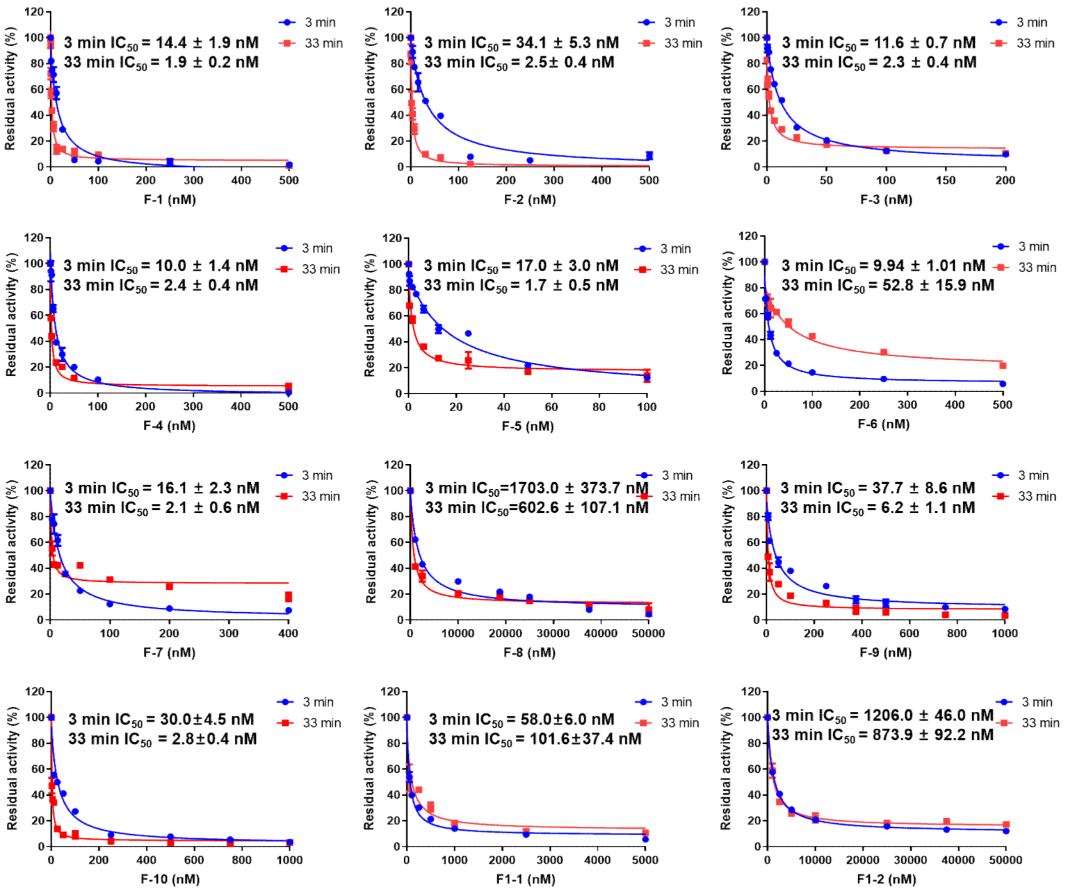

Fig. S6. Inhibitory activities of the halogenated fragment library against hCES1A. Data are reported as the mean ± SD (n = 3 repeats).

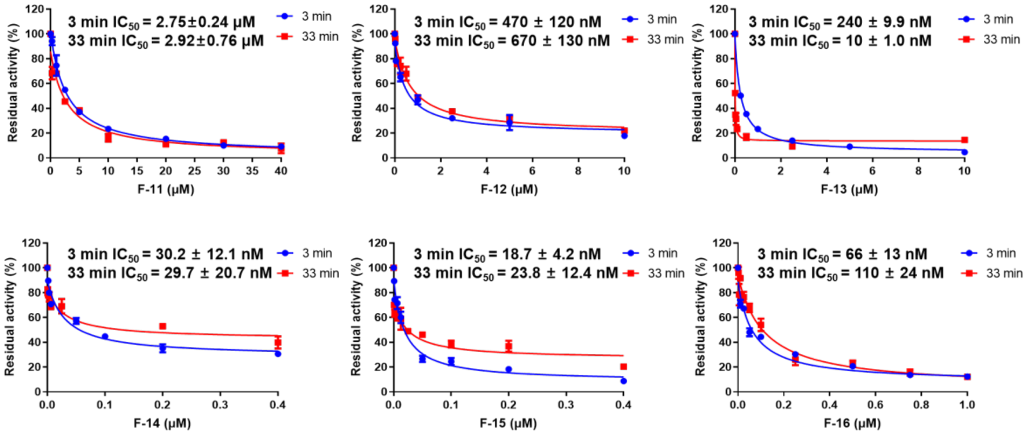

Fig. S7. Chemical structures and inhibitory activities against hCES1A of derivatives **F-11** to **F-16**. Data are reported as the mean ± SD (n = 3 repeats).

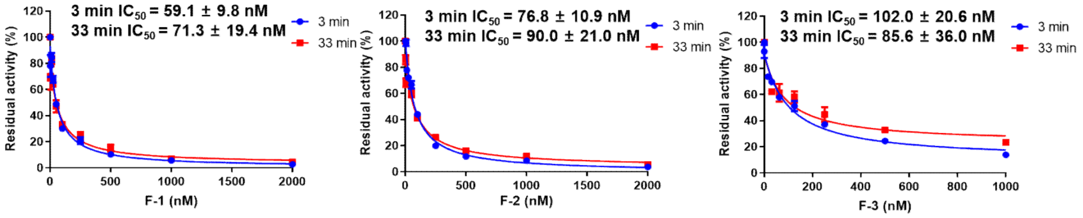

Fig. S8. Inhibitory activities of the halogenated fragment library against hCES2A. Data are

reported as the mean ± SD (n = 3 repeats).

Table S1. Calculated HOMO and LUMO energies for the 'capped' methyl acetylserinate and different trifluoroacetyl fragments, respectively.

**No.**	**HOMO**	**LUMO**	***ΔE*(Ha)**
**serine**	-0.19603	-0.00711	-
**F**	-0.27870	-0.10114	0.09489
**F-1**	-0.27597	-0.10516	0.09087
**F-2**	-0.27208	-0.10705	0.08898
**F-3**	-0.27191	-0.10478	0.09125
**F-4**	-0.27504	-0.11002	0.08601
**F-7**	-0.27094	-0.10566	0.09037
**F-8**	-0.28071	-0.10162	0.09441
**F-9**	-0.26352	-0.11854	0.07749
**F-10**	-0.27909	-0.11483	0.0812

Calculations employed the the rDFT/6-31+G (d,p) level of theory in the Gaussian09 suite of programs. Geometries were optimised and frequencies computed to verify that they are minima. *ΔE*=*E*(LUMO of trifluoroacetyl fragments)-*E*(HOMO of 'capped' serine)

Table S2. Nano LC-MS/MS analysis of fragments **F-3**, **F-4** binding to serine residues of hCES1A protein which are incubated at 4 °C and 25 °C, respectively. [a]

**Cmpd.**	**4°C**		**25°C**	
	**Coverage (%)**	**Binding sites (Ser)**	**Coverage (%)**	**Binding sites (Ser)**
**F-3**	91.71	n/a	88.71	**228**, 233, 365, 369, 380, 430
**F-4**	92.06	n/a	91.53	14, 21, 22, **228**, 315, 365, 486

[a] Data are collected in parallel.

Table S3. IC_{50} values of the halogenated fragment library against hCES1A and hCES2A.[a]

**Cmpd.**	**R**			**R'**	**hCES1A IC_{50} (nM)**		**hCES2A IC_{50} (nM)**	
	**4-**	**3-**	**2-**		**3 min**	**33 min**	**3 min**	**33 min**
**F**	H	H	H	CF_3	24.1 ± 1.0	11.1 ± 2.0	159.8 ± 18.4	335.7 ± 37.2
**F-1**	Cl	H	H	CF_3	14.4 ± 1.9	1.9 ± 0.2	59.1 ± 9.8	71.3 ± 19.4
**F-2**	H	Cl	H	CF_3	34.1 ± 5.3	2.5± 0.4	76.8 ± 10.9	90.0 ± 21.0
**F-3**	H	H	Cl	CF_3	11.6 ± 0.7	2.3 ± 0.4	102.0 ± 20.6	85.6 ± 36.0
**F-4**	Cl	Cl	H	CF_3	10.0 ± 1.4	2.4 ± 0.4	-	-
**F-5**	H	Br	H	CF_3	17.0 ± 3.0	1.7 ± 0.5	-	-
**F-6**	H	H	Br	CF_3	9.9 ± 1.01	52.8 ± 15.9	-	-
**F-7**	Br	H	H	CF_3	16.1 ± 2.3	2.1 ± 0.6	-	-
**F-8**	F	H	H	CF_3	1703.0 ± 373.7	602.6 ± 107.1	-	-

**F-9**	I	H	H	CF_3	37.7 ± 8.6	6.2 ± 1.1	-	-
**F-10**	Cl	Cl	Cl	CF_3	30.0±4.5	2.8±0.4	-	-
**F-11**	CF_3	H	H	CF_3	>2000	>2000	-	-
**F-12**	NH_2	H	H	CF_3	470±120	670±130	-	-
**F-13**	$N(CH_3)_2$	H	H	CF_3	240±9.9	10±1.0	-	-
**F-14**	OMe	H	H	CF_3	30.2±12.1	29.7±20.7	-	-
**F-15**	Et	H	H	CF_3	18.7±4.2	23.8±12.4	-	-
**F-16**	naphthalene			CF_3	66±13	110±24	-	-
**F1-1**	Cl	H	H	CHF_2	58.0±6.0	101.6±37.4	-	-
**F1-2**	Cl	H	H	CH_2F	1206.0 ± 46.0	873.9 ± 92.2	-	-

[a] Data are reported as the mean ± SD (n = 3 repeats).

Table S4. X-ray crystallography data collection and refinement statistics for Compound **F-3**.

**Data collection**	
Data set	F-3
Wavelength (Å)	0.979
Resolution (Å)	92.83-1.89 (2.00-1.89)
Space group	R 3 2
Cell dimensions (Å/degree)	110.2 110.2 278.5<br>90.0 90.0 120.0
Unique reflections	50,889 (6465)
Completeness (%)	97.7 (85.9)
Rmeas (%)	17.2 (202.7)
Rpim (%)	4.5 (63.9)
Redundancy	14.0 (9.0)
Average *I*/σ(I)	13.0 (1.5)
Wilson B value ($Å^2$)	7.48
**Statistics for Refinement**	
Resolution (Å)	47.18-1.89 (1.96-1.89)
Rwork (%)	19.5 (32.4)
Rfree (%)	22.7 (32.1)
Reflections used	52267 (4624)
R.m.s.d.	
Bond (degree)	1.170
Length (Å)	0.010
No. of atoms	4,703

Protein	4,139
Ligand	13
Water	551
Average B factors (Å^2)	14.63
Protein	13.24
Ligand	18.26
Water	25.05
Ramachandran plot	
Favored region (%)	96.80
Allowed region (%)	3.20
Outliers (%)	0

Table S5. X-ray crystallography data collection and refinement statistics for Compound **F-4**.

**Data collection**	
Data set	F-4
Wavelength (Å)	0.979
Resolution (Å)	78.83-1.83 (1.93-1.83)
Space group	R 3 2
Cell dimensions (Å/degree)	110.5 110.5 278.3<br>90.0 90.0 120.0
Unique reflections	57,928 (8371)
Completeness (%)	100.0 (100.0)
Rmeas (%)	20.5 (261.0)
Rpim (%)	5.7 (80.4)
Redundancy	12.5 (9.8)
Average $I/\sigma(I)$	14.6 (3.3)
Wilson B value (Å^2)	18.00
**Statistics for Refinement**	
Resolution (Å)	32.08-1.83 (1.90-1.83)
Rwork (%)	17.4 (27.9)
Rfree (%)	18.6 (28.7)
Reflections used	57812 (5628)
R.m.s.d.	
Bond (degree)	1.130
Length (Å)	0.009
No. of atoms	4,711
Protein	4,139
Ligand	15

Water	557
Average B factors (Å^2)	23.84
Protein	22.53
Ligand	24.89
Water	33.49
Ramachandran plot	
Favored region (%)	97.36
Allowed region (%)	2.64
Outliers (%)	0

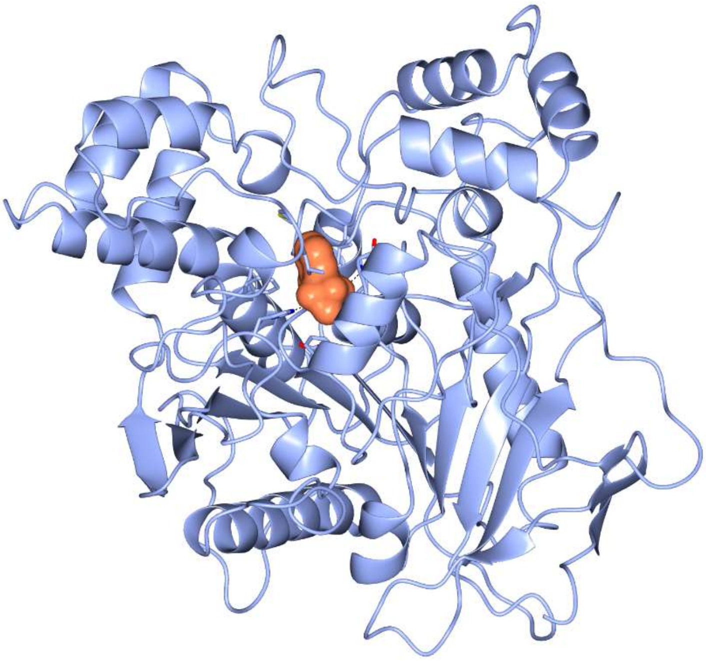

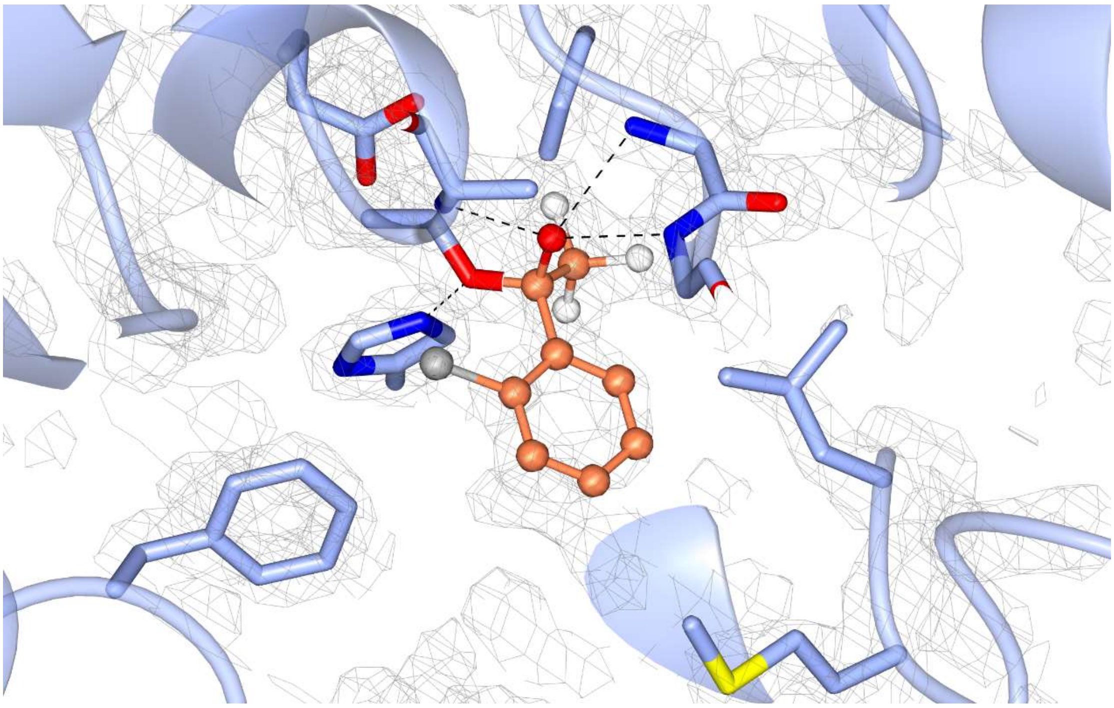

Fig. S9. Dose-response crystal soaks of **F-3** (coral ball and stick) into hCES1A (ice blue ribbons). Electron density of bound ligand is shown in grey mesh. Hydrogen bonding interactions are highlighted in black dotted lines. Water molecules are shown as red spheres. Structure determined by Dr Shilong Fan (Technology Center for Protein Sciences, Tsinghua University) at a resolution of 1.83 Å. The image generated using CCP4MG. Data are deposited at the PBD under D_1300050786 and D_1300050748, respectively. The coordinate data would be obtained from https://www.rcsb.org/ at the time of publication.

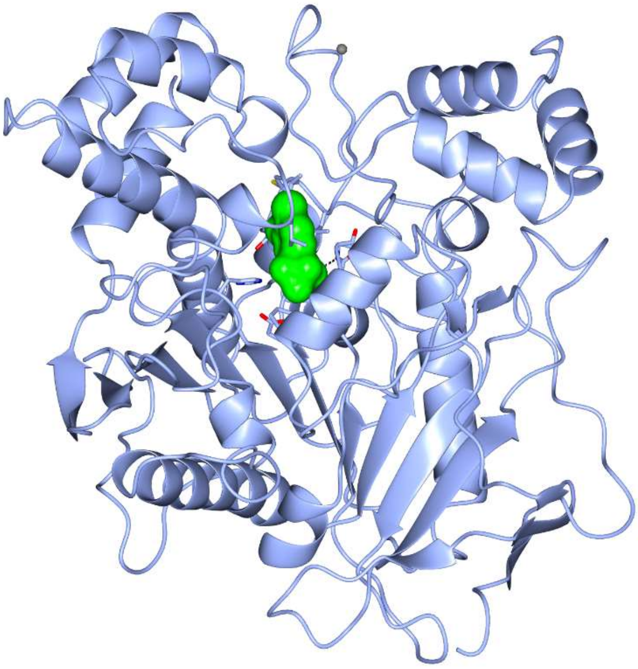

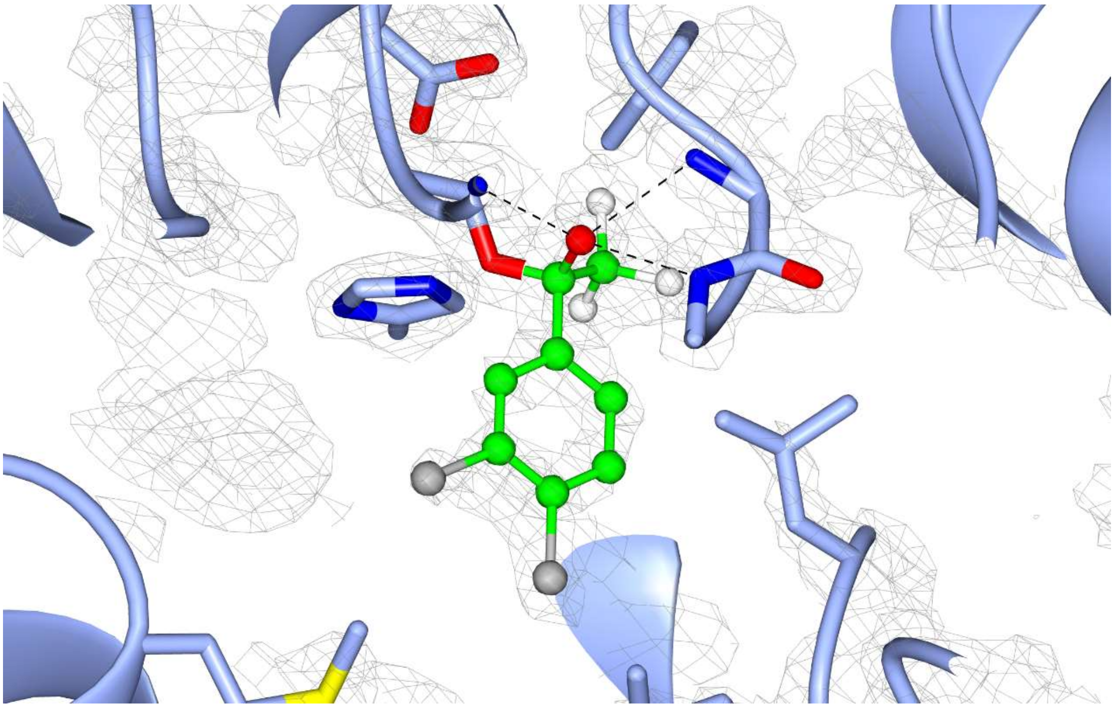

Fig. S10. Dose-response crystal soaks of **F-4** (green ball and stick) into hCES1A (ice blue ribbons). Electron density of bound ligand is shown in grey mesh. Hydrogen bonding interactions are highlighted in black dotted lines. Water molecules are shown as red spheres. Structure

determined by Dr Shilong Fan (Technology Center for Protein Sciences, Tsinghua University) at a resolution of 1.83 Å. The image generated using CCP4MG. Data are deposited at the PBD under D_1300050786 and D_1300050748, respectively. The coordinate data would be obtained from https://www.rcsb.org/ at the time of publication.

3. Reference

1. M. J. Frisch, G. W. T., H. B. Schlegel, G. E. Scuseria, M. A. Robb, J. R. Cheeseman, G. Scalmani, V. Barone, G. A. Petersson, H. Nakatsuji, X. Li, M. Caricato, A. Marenich, J. Bloino, B. G. Janesko, R. Gomperts, B. Mennucci, H. P. Hratchian, J. V. Ortiz, A. F. Izmaylov, J. L. Sonnenberg, D. Williams-Young, F. Ding, F. Lipparini, F. Egidi, J. Goings, B. Peng, A. Petrone, T. Henderson, D. Ranasinghe, V. G. Zakrzewski, J. Gao, N. Rega, G. Zheng, W. Liang, M. Hada, M. Ehara, K. Toyota, R. Fukuda, J. Hasegawa, M. Ishida, T. Nakajima, Y. Honda, O. Kitao, H. Nakai, T. Vreven, K. Throssell, J. A. Montgomery, Jr., J. E. Peralta, F. Ogliaro, M. Bearpark, J. J. Heyd, E. Brothers, K. N. Kudin, V. N. Staroverov, T. Keith, R. Kobayashi, J. Normand, K. Raghavachari, A. Rendell, J. C. Burant, S. S. Iyengar, J. Tomasi, M. Cossi, J. M. Millam, M. Klene, C. Adamo, R. Cammi, J. W. Ochterski, R. L. Martin, K. Morokuma, O. Farkas, J. B. Foresman, and D. J. Fox *Gaussian 09, Revision A.02*.
2. Wang, D. D.; Zou, L. W.; Jin, Q.; Guan, X. Q.; Yu, Y.; Zhu, Y. D.; Huang, J.; Gao, P.; Wang, P.; Ge, G. B.; Yang, L., Bioluminescent Sensor Reveals that Carboxylesterase 1A is a Novel Endoplasmic Reticulum-Derived Serologic Indicator for Hepatocyte Injury. *ACS Sens* **2020,** *5* (7), 1987-1995.
3. Huo, P. C.; Guan, X. Q.; Liu, P.; Song, Y. Q.; Sun, M. R.; He, R. J.; Zou, L. W.; Xue, L. J.; Shi, J. H.; Zhang, N.; Liu, Z. G.; Ge, G. B., Design, synthesis and biological evaluation of indanone-chalcone hybrids as potent and selective hCES2A inhibitors. *Eur J Med Chem* **2021,** *209*, 112856.
4. Qin, X. Y.; Hou, X. D.; Zhu, G. H.; Xiong, Y.; Song, Y. Q.; Zhu, L.; Zhao, D. F.; Jia, S. N.; Hou, J.; Tang, H.; Ge, G. B., Discovery and Characterization of the Naturally Occurring Inhibitors Against Human Pancreatic Lipase in Ampelopsis grossedentata. *Frontiers in nutrition* **2022,** *9*, 844195.
5. Zhao, Y.; Jolly, S.; Benvegnu, S.; Jones, E. Y.; Fish, P. V., Small-molecule inhibitors of carboxylesterase Notum. *Future medicinal chemistry* **2021,** *13* (11), 1001-1015.
6. Fang, C.-M.; Ku, M.-C.; Chang, C.-K.; Liang, H.-C.; Wang, T.-F.; Wu, C.-H.; Chen, S.-H., Identification of Endogenous Site-specific Covalent Binding of Catechol Estrogens to Serum Proteins in Human Blood. *Toxicological sciences : an official journal of the Society of Toxicology* **2015,** *148*.
7. Otwinowski, Z.; Minor, W., [20] Processing of X-ray diffraction data collected in oscillation mode. In *Methods in Enzymology*, Academic Press: 1997; Vol. 276, pp 307-326.
8. Adams, P. D.; Afonine, P. V.; Bunkoczi, G.; Chen, V. B.; Davis, I. W.; Echols, N.; Headd, J. J.; Hung, L.-W.; Kapral, G. J.; Grosse-Kunstleve, R. W.; McCoy, A. J.; Moriarty, N. W.; Oeffner, R.; Read, R. J.; Richardson, D. C.; Richardson, J. S.; Terwilliger, T. C.; Zwart, P. H., PHENIX: a comprehensive Python-based system for macromolecular structure solution. *Acta Crystallographica Section D* **2010,** *66* (2), 213-221.
9. Emsley, P.; Cowtan, K., Coot: model-building tools for molecular graphics. *Acta Crystallographica Section D* **2004,** *60* (12 Part 1), 2126-2132.
